# Mental health during and after pregnancy in medically assisted reproduction: a danish cohort study

**DOI:** 10.1007/s00737-024-01553-y

**Published:** 2025-01-10

**Authors:** Marie Mulvad Grønlund, Line Riis Jølving, Sören Möller, Rikke Wesselhoeft, Mette Bliddal

**Affiliations:** 1 Research Unit OPEN, Department of Clinical Research, University of Southern Denmark, JP Winsløw Vej 21, Odense, DK - 5000 Denmark; 2 Center for Clinical Epidemiology, Odense Denmark and Research Unit of Clinical Epidemiology, Department of Clinical Research, Odense University Hospital, University of Southern Denmark, Odense, Denmark; 3 Clinical Pharmacology, Pharmacy, and Environmental Medicine, Department of Public Health, University of Southern Denmark, Odense, Denmark; 4 Research Unit of Child and Adolescent Psychiatry Southern Denmark, Odense, Denmark

**Keywords:** Infertility, Medically assisted reproduction, Mental health, PSS-10, EPDS

## Abstract

**Purpose:**

Infertility is common and an increasing number of women go through medically assisted reproduction (fertility treatment) to achieve pregnancy. This may affect mental health. We examined if fertility treatment and the specific fertility treatment method used (in vivo or in vitro) were associated with impaired mental health during or after pregnancy.

**Methods:**

Using self-reported data from the Odense Child Cohort, we assessed prenatal stress by the 10-item Cohen Perceived Stress Scale (PSS-10) during pregnancy at median gestational week 27 and postnatal depressive symptoms by the Edinburgh Postnatal Depression Scale (EPDS) at median postpartum week 15. We compared fertility-treated women overall and by fertility treatment method to women who conceived spontaneously. We conducted linear regression analyses to evaluate the PSS-10 score dimensionally and logistic regression to evaluate EPDS scores above cut-off (≥ 11).

**Results:**

A total of 108 of 820 women (13%) gave birth after fertility treatment. Their prenatal mean stress (PSS-10) score was 11.38 compared to 11.78 for women who conceived spontaneously, leading to an adjusted mean difference of -0.09 points (95% confidence interval (CI): -1.88 to 1.69). In the fertility-treated group, 9.7% had EPDS scores ≥ 11 compared to 10.7% in the spontaneous conception group (adjusted odds ratio of 0.71 (95% CI: 0.26 to 1.91)). The MAR method (in vivo/*vitro*) did not influence these results.

**Conclusion:**

Women who gave birth after fertility treatment did not report higher levels of prenatal stress or postpartum depressive symptoms than women who conceived spontaneously.

## Article highlights

In a Danish cohort of new mothers, 108 of 820 (13%) gave birth after medically assisted reproduction.

The women prospectively filled validated questionnaires on mental health during and after pregnancy.

Medically assisted reproduction did not affect the risk of prenatal stress or postpartum depression symptoms.

## Introduction

Up to 18% of all couples experience infertility during their reproductive life span (Cox et al. 2022). The number of children conceived by medically assisted reproduction (fertility treatment) is increasing (De Geyter et al. 2020), and in 2019, one in every 10 children born in Denmark was conceived after fertility treatment (Fertilitetsselskab 2019).

Becoming a mother is life-changing and the transition into motherhood is often difficult (Hansen et al., 2021). As a result, women may experience psychological vulnerability during the perinatal period with an increased risk of developing or worsening mental disorders (Howard and Khalifeh 2020). Infertility may add to this risk, as women with reduced fertility report more depressive symptoms (Herbert et al. 2010) and psychological distress compared to fertile women (Lansakara et al. 2011). On top of that, fertility treatment is stressful and usually characterized by somatic complaints and uncertainty that may last for months or even years. This existential crisis in life may trigger symptoms of stress and depression (Boivin 2019).

Few studies have examined if fertility treatment leads to stress or depressive symptoms during pregnancy or in the postpartum period in women with successful pregnancies. Also, results are conflicting with one study showing elevated levels of stress in fertility-treated women during pregnancy (Hjelmstedt et al. 2003). Raguz et al., however, found lower stress levels in fertility-treated women compared to women with spontaneous pregnancies (Raguz et al. 2014). Studies examining postpartum depressive symptoms have generally shown similar levels in fertility-treated women as women who conceive spontaneously, but this has not been investigated for Danish women (Chen et al. 2019; Gambadauro et al. 2017; Gressier et al. 2015; Salih Joelsson et al. 2017). Further, no studies have examined depressive symptoms across specific fertility treatment methods. It is likely that more invasive procedures such as in vitro fertilization (IVF) and intra cytoplasmic sperm injection (ICSI), could impact mental health to a larger degree than less invasive procedures like intrauterine insemination (IUI). With the increasing number of women going through fertility treatment, it is important to improve our understanding of their mental well-being during this emotionally and physically challenging time in life. This cohort study aimed to examine the association between fertility treatment overall and by fertility treatment methods and mental health. Using data from the Odense Child Cohort (OCC) we examined if women who gave birth after fertility treatment reported higher stress levels during pregnancy or depressive symptoms after birth than women who conceived spontaneously.

## Methods

Using self-reported prospectively collected information from 820 women from OCC, we conducted a cohort study examining perceived stress during pregnancy and postpartum depressive symptoms according to the way of conception overall and by fertility treatment method.

### Database and study population

We used data from the OCC, an ongoing prospective birth cohort that recruited pregnant women from the Municipality of Odense, Denmark, between 2010 and 2012. A total of 2550 pregnant women initially accepted participation in the cohort (Kyhl et al. 2015).

We used questionnaire data filled during pregnancy and 15 weeks postpartum (median 15 weeks, interquartile range 13–17). The questionnaires included baseline information on socio-demography, women’s weight and height, way of conception, fertility treatment method, history of reproductive system disease, time to pregnancy, and validated screening questionnaires on perceived stress and postpartum depressive symptoms.

We included all women from OCC who had a livebirth and gave information on the method of conception and completed the stress- and/or depression screening questionnaires.

### Exposure

We defined women as going through fertility treatment if they replied confirmatively to have received help to conceive. All others were considered to have conceived spontaneously. We further divided fertility treated women into two fertility treatment method groups: in vivo (including IUI) and in vitro (including IVF and ICSI). Women who indicated both IUI and IVF/ICSI were classified as in vitro.

### Outcome

Women in the OCC filled the Cohen’s Perceived Stress Scale (PSS-10) at gestational week 27 (median 27 weeks, interquartile range 27–28) (Cohen et al. 1983). The PSS-10 is a self-report 10-item screening questionnaire, which is considered to have high validity (Lee 2012). Each question refers to the experience of a specific stress symptom over the last four weeks and the 10 items are rated on a five-point Likert Scale (0 through 4). Total scores range from 0 to 40 and are obtained by reversing positive items and summing all 10 items. Higher scores indicate higher levels of perceived stress.

Maternal depressive symptoms were assessed 15 weeks postpartum (median 15 weeks, interquartile range 13–17) using the Edinburgh Postnatal Depression Scale (EPDS) (Cox et al. 1987). The EPDS is a self-report 10-item screening questionnaire where each question refers to specific depressive symptoms over the last seven days. It is rated on a four-point Likert Scale (0 through 3) providing a total score range from 0 to 30. A high level of postnatal depression symptoms was defined as having a total score *≥* 11, as this cut-off has been found to maximize the combined sensitivity and specificity (Levis et al. 2020).

### Covariates

The following a priori selected variables were included as covariates from the OCC questionnaires: Maternal age at childbirth in OCC (continuous), body mass index (BMI) (underweight (< 18.5), normal weight (18.5–24.9) and overweight (*≥* 25) (WHO 2000), maternal smoking during pregnancy (yes/no), maternal alcohol consumption during pregnancy (yes/no), highest level of education (lower (high school or less), intermediate (high school + 1–3 years), and higher (high school ≥ 4 years), civil status (married/cohabitation/divorced or separated/single), time period from conception intention to pregnancy (0–5 month/6–12 month/>12 month/didn’t try/don’t know), and parity (0/≥1). We further included any history of self-reported depression (yes/no) and any history of a reproductive system disease (yes/no) (defined as, any of the following: ovarian-infection or cyst, endometriosis, or polycystic ovarian syndrome). The covariates were chosen based on clinical insight and directed acyclic graphs (Tennant et al. 2021).

### Statistical analysis

Descriptive statistics were performed to describe differences in demographics and clinical characteristics between fertility treated women and women who conceived spontaneously and by treatment method. Analyses were performed for fertility treatment overall and by treatment method (in vivo or in vitro). The group with spontaneous conceptions was used as a reference.

A considerable number of women in the OCC did not complete the PSS-10 and/or EPDS, and we therefore compared demographic and clinical characteristics between women included in our study population and non-responders to address potential selection bias. We compared the responders and the non-responders using two-sample t-tests for continuous data or Fisher’s exact tests for categorical data.

We compared the level of maternal stress based on the discrete numerical sum of the PSS-10 score between the two groups by estimating crude and adjusted coefficients using linear regression analyses with 95% confidence intervals (95% CI).

We compare the number of individuals with postpartum depressive symptom scores above the cut-off (dichotomized at EPDS *≥* 11) by calculating crude and adjusted odds ratio (OR) using logistic regression analyses. We adjusted the analyses for the abovementioned covariates. The analyses were conducted using Stata, version 17.

## Results

**Fig. 1:**
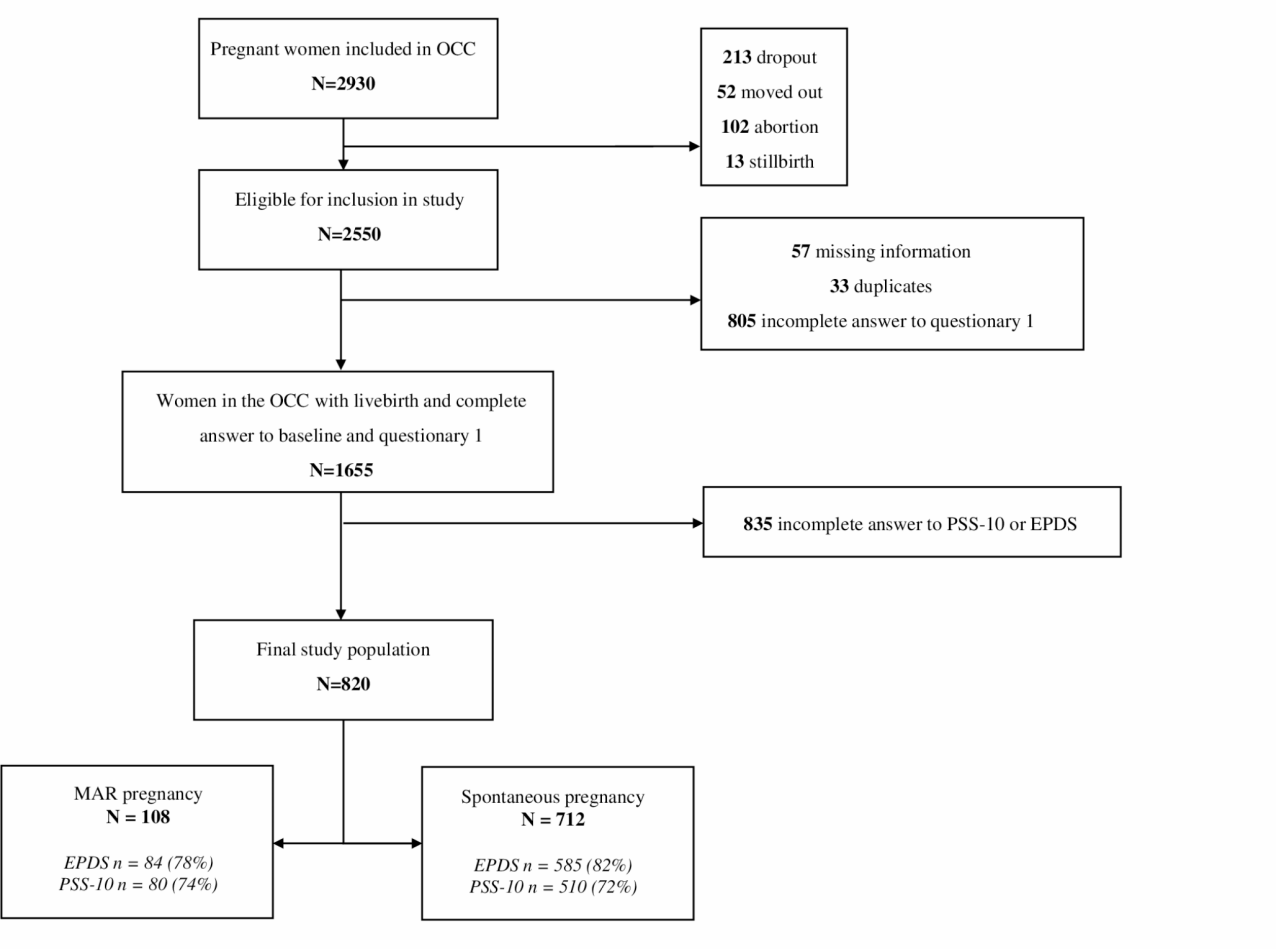
Flow chart for the included 820 women from Odense Child Cohort (OCC)

Of the 2,550 women included in the OCC, the final study population consisted of 820 women. Of these, 108 (13%) had conceived after fertility treatment, and 712 (87%) obtained pregnancy spontaneously (Fig. 1). Among women undergoing fertility treatment, the distribution of in vitro versus in vivo fertilization was 49% (*N* = 53) in each group, and the method for the remaining 2% (*N* = 2) was undetermined.

### Baseline characteristics of the study population

Compared to women who conceived spontaneously, fertility-treated women were older, were more likely to be first-time mothers and of normal weight, and were less likely to smoke (Table 1). These women had higher educational levels and were more often single than women who conceived spontaneously. Fertility-treated women had a four-fold risk of previous reproductive system disease compared to women who conceived spontaneously (40% vs. 9.6%). Reflecting this, seven out of 10 in the fertility-treated group had tried to become pregnant for more than 12 months compared to less than 1 of 10 in women who conceived spontaneously (Table 1). When stratifying on treatment method, women treated using in vitro methods were older and more often nulliparous, had a history of reproductive system disorders and longer waiting time to conception than women in in vivo treatment (Online resource 1).

**Table 1:** Baseline characteristics of the women conceived spontaneously and using medically assisted reproduction (MAR) in the Odense child cohort, Denmark (*N* = 820)

Characteristics		Spontaneous conception women	MAR conception women
(*N* = 820)		(*N* = 712)	(*N* = 108)
Maternal age (years) mean at inclusion (SD)		30.1 (4.3)	31.9 (4.5)
Parity (n (%))			
	0	404 (57)	83 (77)
	*≥* 1	308 (43)	25 (23)
BMI (n (%))			
	Underweight (< 18,5)	19 (2.7)	5 (4.6)
	Normal weight (18,5–25)	452 (64)	64 (59)
	Overweight (> 25)	241(34)	39 (36)
Smoking during pregnancy (n (%))			
	No	689 (97)	> 103
	Yes	23 (3.2)	< 5 ^a^
Alcohol consumption during pregnancy (n (%)) ^b^			
	No	643 (93)	99 (94)
	Yes	50 (7.2)	6 (5.7)
Education level (n (%))			
	Lower	150 (21)	15 (14)
	Intermediate	263 (37)	34 (32)
	Higher	296 (42)	59 (55)
Civil status (n (%))			
	Married	289 (41)	49 (45)
	Cohabitation	414 (58)	50 (46)
	Separated/single	9 (1.3)	9 (8.3)
History of reproductive system disease (n (%)) ^c^			
	No	625 (90)	62 (60)
	Yes	66 (9.6)	41 (40)
Time to pregnancy (n (%))			
	0–5 months	518 (73)	13 (12)
	6–12 months	111 (16)	18 (17)
	> 12 months	56 (7.9)	77 (71)
	Didn’t try	20 (2.8)	0 (0.0)
	Don’t know	7 (1.0)	0 (0.0)
History of depression (n (%))			
	No	596 (86)	87 (85)
	Yes	96 (14)	16 (16)

*SD* standard deviation, *BMI* body mass index.

^a^ Due to regulations not allowing to report explicit numbers below 5

^b^ Asked about alcohol consumption within the last 14 days in 10–26 weeks of pregnancy.

^c^ Ovarian infection- and cyst, endometriosis, polycystic ovaries syndrome.

Of the 1,655 women who completed the baseline questionnaire, 50.5% (*n* = 835) did not fill out the PSS-10 or the EPDS (Fig. 1). Non-responders differed from the study population in being more likely to smoke (*p* = 0,02), drink alcohol during pregnancy (*p* < 0.001), have lower educational status(*p* < 0.001), and being less likely to suffer from reproductive disorders (*p* = 0.019) compared to responders (**Online resource 2**). To be noted, the non-responder population had 17.2% missing data, making it challenging to draw any direct comparisons with the study population.

### Perceived stress during pregnancy

The mean PSS-10 score was 11.26 (standard deviation (SD) 5.54). Fertility-treated women had a mean PSS-10 score of 11.38 (standard deviation (SD) 5.05) compared to 11.24 (SD 5.61) in women who conceived spontaneously. The levels of perceived stress did not differ between the two groups, yielding a mean adjusted difference of 0.09 (95% CI: -1.88 to 1.69) i.e. the women who conceived using MAR had a 0.009 lower mean score in the PSS-10 compared to women who conceived spontaneously (Table 2). When stratifying on fertility treatment method, the in vivo group had a mean score of 11.78 (SD 5.34), and the in vitro group had a mean score of 10.97 (SD 4.84). When comparing to women who conceived spontaneously, there was no difference between scores with an adjusted mean difference of 0.28 points (95% CI: -1.82 to 2.37) in the in vivo group and − 0.59 (95% CI: -2.98 to 1.81) in the in vitro group.

**Table 2:** Crude and adjusted linear regression analyses and 95% CI for perceived stress symptoms (total PSS-10 score) perinatal in relation to mode of conception and by type of medically assisted reproduction (MAR) treatment (*N* = 590)

			PSS-10 score	Coefficient (95% CI)			
		n	Mean (SD)	Unadjusted	*P* Value	Adjusted*	*P* Value
Spontaneous conception		510	11.24 (5.61)	1.0		1.0	
MAR conception		80	11.38 (5.05)	0.14 (-1.17;1.44)	0.84	-0.09 (-1.88; 1.69)	0.92
	In vivo^*a*^	40	11.78 (5.34)	0.54 (-1.25; 2.33)	0.56	0.28 (-1.82;2.37)	0.80
	In vitro^*b*^	39	10.97 (4.84)	-0.27 (-2.08;1.55)	0.77	-0.59 (-2.98;1.81)	0.63

PSS-10: Perceived Stress Scale, CI: confidence interval, MAR Medically assisted reproduction,

^a^In vivo (HSG, IUI). ^b^In vitro (IVF, ICSI)

Total PSS-10 score range from 0–40

*Adjusted for: age, BMI, smoking during pregnancy, alcohol consumption during pregnancy, education level,

time period to pregnancy, civil status, history of depression, history of reproductive system disease, and parity

### Depressive symptoms after birth

Regarding depressive symptoms after birth, 9.7% of fertility-treated women had an EPDS score ≥ 11 compared to 11% in women who conceived spontaneously yielding a 0.71 adjusted odds (95% CI: 0.26 to1.91) for postpartum depressive symptoms in treated women compared to untreated women. (Table 3).

**Table 3:** Crude and adjusted odds ratio and 95% CI for high risk of postpartum depressive symptoms (EPDS *≥* 11) in relation to mode of conception and by type of medically assisted reproduction (MAR) treatment (*N* = 748)

		EPDS ≥ 11	EPDS < 11	Odds ratio (95% CI)			
		n (%)	n (%)	Unadjusted	*P* Value	Adjusted*	*P* Value
Spontaneous conception		70 (11)	585 (89)	1.0		1.0	
MAR conception		9 (9.7)	84 (90)	0.90 (0.43;1.86)	0.77	0.71 (0.26;1.91)	0.50
	In vivo^a^	4 (9.8)	37 (90)	0.93 (0.31;2.61)	0.85	0.78 (0.24;2.60)	0.69
	In vitro^b^	5 (10)	45 (90)	0.12 (0.36;2.42)	0.88	0.66 (0.18;2.44)	0.53

EPDS: Edinburgh postnatal depression score, CI: confidence interval, MAR: Medically assisted reproduction

^a^In vivo (HSG, IUI). ^b^In vitro (IVF, ICSI)

*Adjusted for: age, BMI, smoking during pregnancy, alcohol consumption during pregnancy, education level,

time period to pregnancy, civil status, history of depression, history of reproductive system disease and parity

**Fig. 2:**
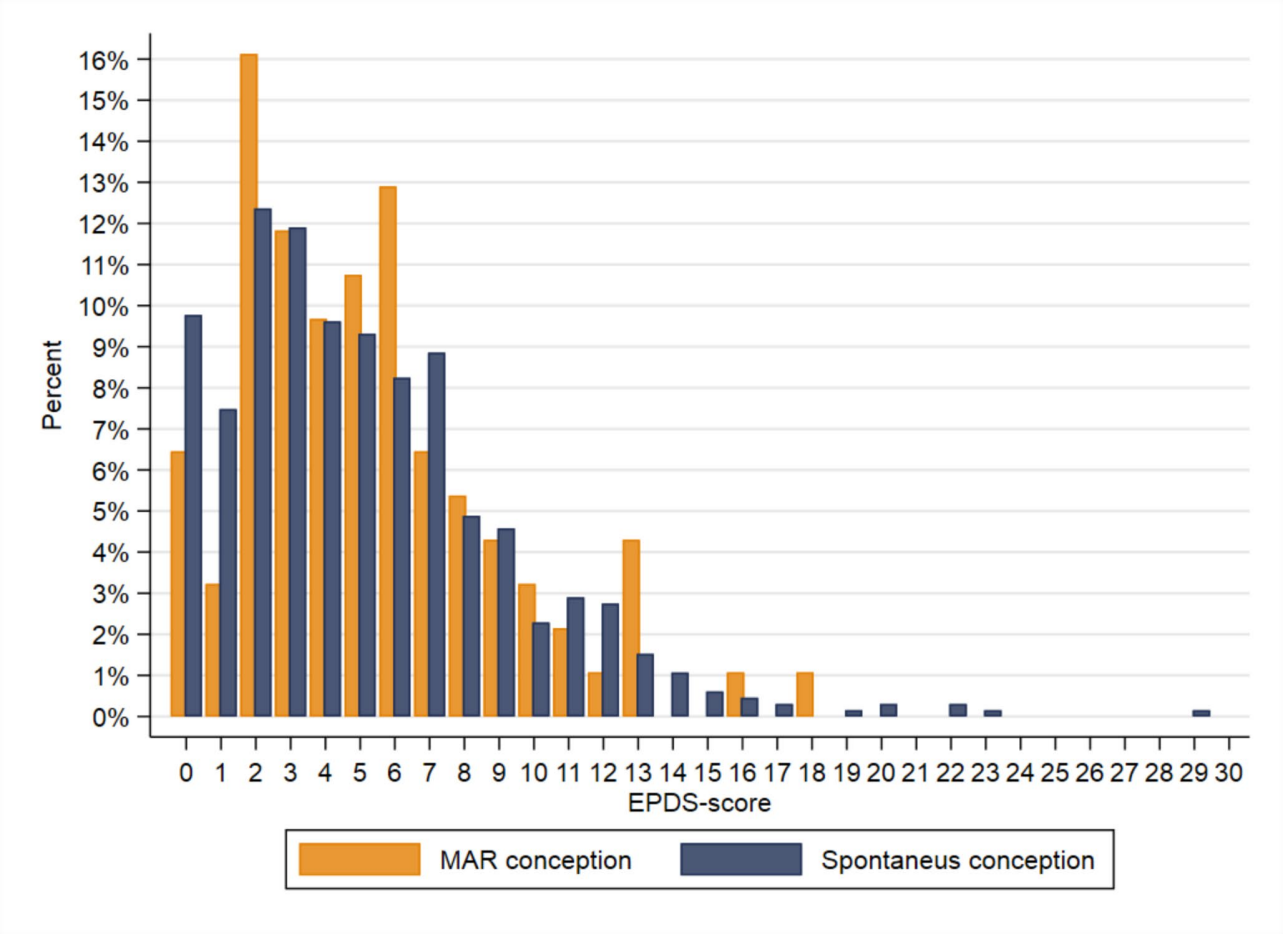
Bar chart illustrating the distribution of EPDS-score in women conceived spontaneous (*n* = 585) and using medically assisted reproduction (MAR) (*n* = 84)

When stratified by fertility treatment method, EPDS scores ≥ 11 were reported by 9.8% of the in vivo group and by 10% of the in vitro group. The in vivo- and in vitro groups had adjusted ORs of 0.78 (CI: 0.24 to 2.60) and 0.66 (CI: 0.18 to 2.44), respectively, compared to the women who conceived spontaneously. The bar chart (Fig. 2) further demonstrates this result, with the bars representing the total EPDS score in women in fertility treatment overall and women who conceived spontaneously.

## Discussion

In this Danish cohort study, women pregnant after medically assisted reproduction (fertility treatment) reported the same level of prenatal stress and depressive symptoms as women who conceived spontaneously. The fertility treatment method did not impact the results. Our findings are reassuring given the increasing rates of fertility treatment and the importance of maternal well-being for both the newborn child and the family.

Previous studies examining stress related to fertility treatment show conflicting results (Hjelmstedt et al. 2003; Raguz et al. 2014). Hjelmstedt et al. found that fertility-treated women were more anxious about losing the pregnancy compared to women with spontaneous pregnancies (Hjelmstedt et al. 2003). Conversely, a Canadian study found lower levels of stress during pregnancy in women who conceived after fertility treatment compared to women with spontaneous conception (Raguz et al. 2014). Our findings align with the results of the two referenced studies, indicating no association between fertility treatment and high stress levels. Differences in results can be due to different study populations and health care systems, and can potentially also be explained by differences in cut-offs of high- and low-stress indicators. Consistent with our results, meta-analyses have also failed to demonstrate a significantly increased risk of postnatal depressive symptoms among fertility-treated women (Chen et al. 2019; Gressier et al. 2015).

Our cohort of women in fertility treatment represent women who were successful in their treatment as was the case in the studies above. An unsuccessful fertility treatment may adversely affect the risk of depression (Kiani et al. 2021), highlighting the importance of future studies examining how unsuccessful fertility treatment influences the mental health of women.

Finally, we did not find differences in stress or depression scores when we stratified by the type of fertility treatment although in vitro fertility treatments may be anticipated to have a greater impact on mental health compared to in vivo methods, owing to their invasiveness and the fact that they are next line treatment after unsuccessful in vivo treatments. However, it is important to note that the small sample sizes and wide confidence intervals in our study may limit the generalizability of these findings.

### Strengths and limitations

Our study benefits from a large study population with a wide range of characteristics available. The use of validated self-report instruments to measure stress- and depression symptoms is a key strength of the study. These questionnaires provide subjective subclinical indications of stress and depressive symptoms, which gives a broader perspective on women’s mental health than data solely from women with clinical diagnoses or a history of medical treatment.

The main limitation is the risk of selection bias. We only included women who provided information on stress or depressive symptoms. Our non-response analysis revealed that OCC women who did not reply differed from the study population on factors that might be associated with mental health indicators such as smoking and alcohol consumption. Additionally, women with more severe indications of stress and/or depression may have omitted to fill the questionnaires due to lack in energy or motivation required to respond to the sensitive questions. Consequently, these women might not have been included in the analyses. We cannot rule out that this selection bias may affect our results. Numbers of fertility treatment cycles as well as time to obtain pregnancy may be potential confounders, however, our data set did not hold this information. Due to our sample size, estimates yielded wide confidence intervals, particularly in the analysis stratified by fertility treatment method.

## Conclusion

In this cohort study, we find that Danish women having live-born offspring report the same levels of stress in pregnancy and depressive symptoms after birth, independent of whether their pregnancies were based on fertility treatment or not. Our data also suggests that the invasiveness of fertility treatment does not affect self-reported perinatal mental health. These findings are reassuring, particularly in lights of the increasing rates of fertility treatment and the importance of maternal well-being for the new family.

## Data Availability

Data is not directly available. Permission to gain access can be granted by the Steering Committee of the Odense Child Cohort. Data analysis codes are available upon request.
